# Association of the Expiration of Child Tax Credit Advance Payments With Food Insufficiency in US Households

**DOI:** 10.1001/jamanetworkopen.2022.34438

**Published:** 2022-10-21

**Authors:** Allison Bovell-Ammon, Nicole C. McCann, Martha Mulugeta, Stephanie Ettinger de Cuba, Julia Raifman, Paul Shafer

**Affiliations:** 1 Department of Pediatrics, Boston Medical Center, Boston, Massachusetts; 2 Boston University School of Public Health, Boston, Massachusetts; 3 Boston University School of Medicine, Boston, Massachusetts

## Abstract

**Importance:**

Advance Child Tax Credit (CTC) monthly payments administered to more than 35 million households with children in the US between July and December 2021 were associated with a substantial decrease in food insufficiency. These monthly payments expired in January 2022 after Congress failed to extend the policy, and the subsequent impact on food insufficiency is currently unknown.

**Objective:**

To assess whether the expiration of monthly CTC payments in January 2022 was associated with changes in food insufficiency in US households with children.

**Design, Setting, and Participants:**

This study used repeated cross-sectional, nationally representative data from multiple waves of the Household Pulse Survey, conducted by the US Census Bureau. Online data collection occurred from July 21, 2021, to July 11, 2022, and data analysis was performed in July 2022.

**Exposures:**

The first missed advance CTC payment on January 15, 2022.

**Main Outcomes and Measures:**

The main outcome was unadjusted prevalence of household food insufficiency. Event study specification was used to estimate the association between the expiration of the CTC payments and household food insufficiency with the exposure of being in a household with children present.

**Results:**

The sample (592 044 respondents, representing households with and without children, for a weighted population size of 123 350 770 individuals) was majority female (362 286 individuals [51.3%]) and non-Hispanic White (425 497 individuals [62.2%]), and a plurality of respondents (248 828 [48.3%]) were aged 25 to 44 years at the time of the survey. During the survey wave just before CTC expiration (reference wave, December 29, 2021, to January 10, 2022), unadjusted household food insufficiency was 12.7% among households with children. In late January and early February 2022, following the first missed CTC monthly payment, 13.6% of households with children reported food insufficiency, increasing to 16.0% by late June and early July 2022. The event study specification estimated a 3.2 percentage point increase (95% CI, 1.4-5.0 percentage points; *P* < .001) in food insufficiency by the most recent wave available after the first missed CTC payment (June 29 to July 11, 2022) among households with children compared with the reference wave, a 25% increase.

**Conclusions and Relevance:**

The findings of this study suggest that there was an increase in food insufficiency among households with children after they stopped receiving monthly CTC payments. Given the well-documented associations between inability to afford food and poor health outcomes across the life span, Congress should consider swift action to reinstate this policy.

## Key Points

**Question:**

Was expiration of advance Child Tax Credit (CTC) payments associated with increased food insufficiency in the US?

**Findings:**

In a cross-sectional study of repeated surveys from a nationally representative sample of US households (592 044 respondents), missed CTC monthly payments were associated with a 25% increase in food insufficiency among households with children by July 2022.

**Meaning:**

The findings of this study suggest that the loss of monthly CTC payments was associated with an increase in the prevalence of households with children in US reporting sometimes or often not having enough to eat, a condition associated with adverse health outcomes across the life span.

## Teaser

This cross-sectional study assesses whether the expiration of monthly Child Tax Credit payments in January 2022 was associated with changes in food insufficiency in US households with children.

## Introduction

Difficulty affording enough food is associated with adverse health outcomes among children and adults.^1,2,3,4^ Food insufficiency, a marker for economic strain, is defined by household lack of enough food to eat in the last 7 days.^5^ The related construct of food insecurity—defined as the inability to afford enough food for all family members to live active, healthy lives—has been associated with adverse health outcomes across the life span, particularly during childhood.^1^ National rates of food insufficiency increased in 2020 following the onset of the COVID-19 pandemic and resulting economic crisis. Although overall rates of food insufficiency have fluctuated since 2020, households with children experience consistently higher rates of food insufficiency than families without children.^6^ Given the critically important growth and developmental windows sensitive to nutrient intake and family financial stability during childhood, health experts have warned of the potential lasting effects of increasing economic strain on children and their families.^7,8^

In response to ongoing economic hardship resulting from the COVID-19 pandemic, the US Congress passed several relief packages, including the American Rescue Plan Act (ARPA) in March 2021. One of the largest relief programs in this package was the advance Child Tax Credit (CTC) payments, which was a monthly child allowance to households with children created by an expansion of the existing CTC. Under ARPA, 3 major changes to the credit were enacted for tax year 2021: (1) expansion of eligibility to include families earning very low or no income, (2) a boost in credit amounts from a maximum credit of $2000 per child per year to $3000 per child aged 6 to 17 years per year and $3600 per child younger than 6 years per year, and (3) provision of half of the credit as an advanced monthly payment between July and December 2021 (eTable 1 in the Supplement). As a result of these changes, an estimated 92% of families with children were eligible to receive $250 to $300 monthly per child between July and December 2021.^9,10^ Families who received monthly payments were eligible to claim an additional $1500 to $1800 per child after filing a 2021 tax return. The overwhelming majority of these tax returns were filed during tax season between February and mid-April 2022.^11^ Because of the temporary nature of the ARPA expansion, the advance CTC payments ended in December 2021 and the credit reverted to its original structure and amounts for tax year 2022. Although other federal COVID-19 relief benefits, including pandemic unemployment insurance and eviction protections, expired in fall 2021, the advance CTC was the only major relief benefit to expire in early 2022.

National data show that parents report spending the monthly CTC payments on food, utilities, rent, clothing, and educational expenses.^12^ A growing body of research^13,14,15,16^ indicates that the advance CTC monthly payments were associated with reductions in the child poverty rate and financial hardship among households with children and with improved dietary quality for children. Prior research^10^ using nationally representative data showed a 26% decrease in food insufficiency among households with children after introduction of CTC payments in July 2021. This cross-sectional study examines changes in household food insufficiency following the first missed advance monthly CTC payments in 2022.

## Methods

We used the nationally representative Household Pulse Survey data on demographic characteristics, employment, social supports, and food insufficiency from the US Census Bureau.^17^ We included data from July 21, 2021, to July 11, 2022, to capture the period before and after expiration of the CTC monthly payments. Using an event study specification, we estimated the association between CTC payments and food insufficiency in households with children compared with households without children. The study was exempt from institutional review board approval and the need for informed consent because the data were anonymous and publicly available, in accordance with 45 CFR §46. This study meets the Strengthening the Reporting of Observational Studies in Epidemiology (STROBE) reporting guidelines for cross-sectional studies.^18^

In the Household Pulse Survey, only 1 individual per sampled household is invited to respond, providing responses regarding both their household and themselves. In this analysis, we limited the sample to adults younger than 65 years to capture a working-age population and further restricted the analysis to those without missing data (with the exception of those missing income data, for which we created a missing category). The study period includes data from phases 3.2 to 3.5 of the Household Pulse Survey. The start of the study period, July 21, 2021, is the first survey wave in which the CTC was received by households with children present. The survey wave preceding the exposure—the first missed advance CTC payment on January 15, 2022—was the reference wave (December 29, 2021, to January 10, 2022); all included waves thereafter were after the CTC expiration.

The treated group in this analysis was households with children present, compared with households without children. The outcome of interest was food insufficiency, which was measured according to household-level responses to the following survey item: “Getting enough food can be a problem for some people. In the last 7 days, which of these statements best describes the food eaten in your household?” We defined food insufficiency as a report of “sometimes” or “often” “not [having] enough food to eat” in the past 7 days. Participants who reported having “enough of the kinds of foods [I/we] wanted to eat” or “enough, but not always the kinds of food [I/we] wanted to eat” were considered not to have food insufficiency. These definitions are consistent with prior literature using this data source.^10,19,20^

We report unadjusted rates of food insufficiency among households with and without children in the period before the first missed advance CTC payment (July 21, 2021, through January 10, 2022) and in the period after the first missed advance CTC payment (January 26 through July 11, 2022). In subgroup analysis, we report unadjusted food insufficiency in these periods among households with children that may have been disproportionately impacted by the CTC expiration: (1) low-income households (defined as households reporting annual income <$35 000),^21^ (2) single-adult households (defined as households reporting only 1 adult in the household), and (3) by race and ethnicity (stratified as non-Hispanic White, Hispanic, non-Hispanic Black, non-Hispanic Asian, and another race or ethnicity, which reflects all categories available in public use files [other categories collected but not available in public data because of small numbers]). Race and ethnicity were self-reported by the respondents and were analyzed in this study given the persistent disparities in food insufficiency among families with children and previous analyses suggesting the CTC eligibility expansion disproportionately benefited children in Black and Hispanic families.

### Statistical Analysis

**Figure 1.**
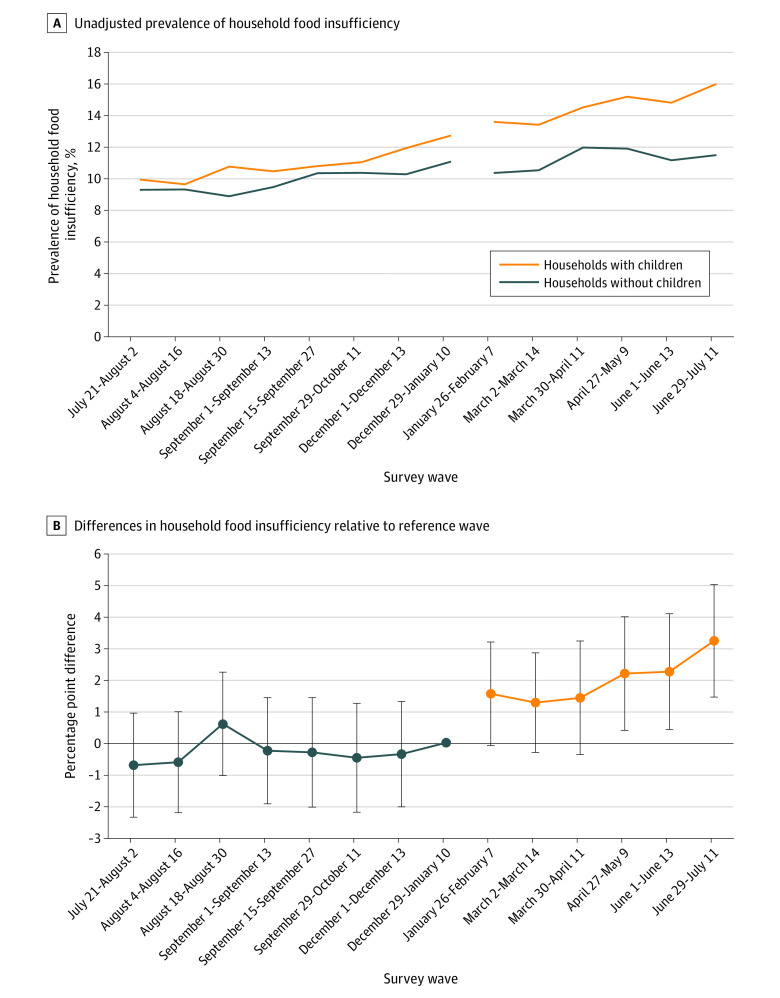
Household Food Insufficiency by Survey Wave for Households With Children Present, US Census Bureau Household Pulse Survey Panel A shows unadjusted prevalence of household food insufficiency in households with children present (targeted by the Child Tax Credit payment, orange lines) compared with households without children present (not targeted by the Child Tax Credit payment, blue lines) by survey wave. Panel B shows marginal effects comparing the differences in household food insufficiency for households with children present compared with those without children present, compared with the difference during the reference wave. In both panels, the reference wave is wave 41, phase 3.3 of the Household Pulse Survey (December 29, 2021 to January 10, 2022), just before the first missed advance Child Tax Credit payment. Error bars represent 95% CIs.

We used an event study specification to estimate the association between the expiration of the CTC payments and household food insufficiency with the exposure of being in a household with children present. An event study is a more flexible version of a traditional difference-in-differences regression model. Although a difference-in-differences regression model relies on treatment and postpolicy indicators and their interaction to identify a differential association, the event study uses survey wave indicators and the interactions between them and the treatment indicator (in this case, households with children present) that accounts for variability in the outcome over time in the postpolicy period. Similar to our prior analysis investigating the outcomes of the start of the CTC,^10^ this approach investigates a natural experiment: expiration of CTC payments in January 2022. This expiration was expected to have a differential impact on households with children present, because only households with children receive the CTC. Event studies rely on the parallel trends assumption, which posits that both during the prepolicy period and the postpolicy period, the comparison groups would have similar outcome trends. In this analysis, the parallel trends assumption held for the pre-CTC expiration period. Before January 10, 2022 (the last survey wave before CTC expiration), there were no significant differences in food insufficiency trends in households with children present compared with households without children present in graphical or statistical evaluation (Figure 1 and eTable 2 in the Supplement).

In the event study specification, covariates included respondent sex at birth (female or male), age group (18-24, 25-44, or 45-65 years), race and ethnicity (non-Hispanic White, Hispanic, non-Hispanic Black, non-Hispanic Asian, or another race or ethnicity), educational level (less than high school, high school or equivalent, some college or 2-year degree, or 4-year degree or more), household income in 2019 (for surveys administered in 2021) or 2020 (for surveys administered in 2022) (<$25,000, $25 000-$34 999, $35 000-$49 999, $50 000-$74 999, $75 000-$149,999, ≥$150,000, or missing), marital status (married or not), number of adults in the household (1, 2, or ≥3), respondent employment in the last 7 days (yes or no), and health insurance coverage (uninsured, public, or private). Participation in and use of other benefit programs were also included as covariates to isolate the outcomes of the advance CTC payments. These additional covariates included unemployment benefits as source of spending or funds by anyone in household in last 7 days (yes or no), Economic Impact Payments (stimulus) as source of spending or funds by anyone in household in last 7 days (yes or no), current participation in Supplemental Nutrition Assistance Program by anyone in the household (yes or no), and receipt of food aid (eg, food pantry) by anyone in the household (yes or no). We also controlled for state fixed effects.

For comparison with our event study specification, we ran a difference-in-differences (a less flexible model with the same exposure and covariates as the event study specification) as an alternative specification. Limited subgroup sample sizes led to underpowered event study estimates since each wave is modeled as a separate association and interaction; thus, we ran difference-in-differences analysis for each subgroup (low-income households, single-adult households, and each race and ethnicity category).

We tested the robustness of our findings by varying model assumptions using 4 alternative models: (1) clustered SEs by state, (2) preperiod restricted to dates after expiration of the federal eviction moratorium and enhanced unemployment benefits (after September 4, 2021), (3) controlling for interaction between unemployment benefits and the presence of children in the household, and (4) controlling for interactions between all included covariates and the presence of children in the household (fully saturated).

In all models, we used US Census–provided household survey weights divided by the 14 waves in our sample. We used 2-sided *t *tests or χ^2^ tests to test for significant differences in characteristics between households with and households without children. *P* < .05 was considered significant. Our analysis was conducted in July 2022 using Stata/MP statistical software version 17 (StataCorp).

## Results

Our weighted sample comprised 592 044 respondents representing households with and without children, for a weighted population size of 123 350 770 individuals. Respondents were majority female (362 286 respondents [51.3%]) and non-Hispanic White (425 497 respondents [62.2%]), with a plurality (248 828 respondents [48.3%]) aged 25 to 44 years at the time of the survey. More than one-third (331 653 respondents [35.2%]) had a 4-year college degree or higher, and nearly one-quarter (171 510 respondents [24.1%]) had an annual household income of $75 000 to $149 000. Weighted individual demographics and household socioeconomic characteristics of our sample are shown in Table 1 for both the full sample and stratified by presence of children in the household.

**Table 1.** Sample Characteristics, Overall and for Households With and Without Children, US Census Bureau Household Pulse Survey, July 2021 to July 2022^a^

Characteristic	Respondents, No. (weighted %)			*P* value
	Full sample (N = 592 044)	Households with children (n = 239 176)	Households without children (n = 352 868)	
Sex at birth				
Female	362 286 (51.3)	152 971 (55.8)	209 315 (48.2)	<.001
Male	229 758 (48.7)	86 205 (44.2)	143 553 (51.8)	
Age group, y				
18-24	21 949 (7.6)	5385 (5.4)	16 564 (9.3)	<.001
25-44	248 828 (48.3)	138 620 (63.2)	110 208 (37.8)	
45-64	321 267 (44.1)	95 171 (31.5)	226 096 (53.0)	
Race and ethnicity				
Hispanic	61 265 (16.2)	29 684 (19.8)	31 581 (13.6)	<.001
Non-Hispanic				
Asian	34 118 (5.4)	15 692 (5.9)	18 426 (5.0)	<.001
Black	46 704 (12.3)	21 279 (14.2)	25 425 (11.0)	<.001
White	425 497 (62.2)	161 715 (55.9)	263 782 (66.6)	<.001
Another race or ethnicity^b^	24 460 (3.9)	10 806 (4.1)	13 654 (3.8)	<.001
Education				
Less than high school	11 876 (6.6)	6373 (8.6)	5503 (5.2)	<.001
High school or equivalent	65 144 (27.6)	25 973 (27.3)	39 171 (27.7)	
Some college or 2-y degree	183 371 (30.7)	72 268 (30.5)	111 103 (30.9)	
4-y Degree or higher	331 653 (35.2)	134 562 (33.6)	197 091 (36.2)	
Marital status				
Married	337 969 (50.2)	170 221 (63.4)	167 748 (40.9)	<.001
Not married	254 075 (49.8)	68 955 (36.6)	185 120 (59.1)	
Health insurance coverage				
Uninsured	61 080 15.2)	25 733 (16.3)	35 347 (14.4)	<.001
Public	60 376 (13.0)	25 772 (14.7)	34 604 (11.7)	
Private	470 588 (71.9)	187 671 (69.0)	282 917 (73.9)	
Respondent employed in the last 7 d	436 079 (69.4)	179 947 (69.2)	256 132 (69.5)	.20
Report of unemployment insurance benefits as spending source in the last 7 d	15 385 (3.5)	6264 (3.8)	9121 (3.3)	<.001
Current participation in Supplemental Nutrition Assistance Program benefits by anyone in the household	52 685 (13.4)	29 212 (19.2)	23 473 (9.3)	<.001
Receipt of food aid in the last 7 d by anyone in the household	24 785 (5.9)	13 175 (8.0)	11 610 (4.5)	<.001
Report of Economic Impact Payment as spending source in the last 7 d	51 246 (11.2)	26 022 (13.9)	25 224 (9.3)	<.001
Adults in household, No.				
1	125 620 (22.0)	32 759 (15.3)	92 861 (26.8)	<.001
2	325 135 (52.0)	147 490 (57.9)	177 645 (47.8)	
≥3	141 289 (26.0)	58 927 (26.8)	82 362 (25.5)	
Children in household, No.				
0	352 868 (58.5)	0	352 868 (100.0)	<.001
1	104 237 (18.0)	104 237 (43.4)	0	
2	87 321 (14.5)	87 321 (35.0)	0	
≥3	47 618 (9.0)	47 618 (21.6)	0	
Annual household income, $				
<25 000	58 453 (14.3)	19 163 (12.9)	39 290 (15.3)	<.001
25 000-34 999	41 974 (9.4)	14 882 (8.8)	27 092 (9.9)	
35 000-49 999	51 476 (10.2)	18 206 (9.3)	33 270 (10.8)	
50 000-74 999	83 276 (14.4)	29 622 (13.2)	53 654 (15.4)	
75 000-149 999	171 510 (24.1)	70 492 (24.0)	101 018 (24.1)	
≥150 000	117 777 (13.3)	54 584 (14.9)	63 193 (12.2)	
Missing	67 578 (14.3)	32 227 (17.0)	35 351 (12.3)	

^a^ Limited to working-age respondents (aged <65 years), weighted using household survey weights divided by the number of survey waves (14). Demographic characteristics are specific to the respondent; receipt of public assistance and other support is either individual or household, as indicated. Two-sided *t* tests or χ^2^ tests were used to test for significant differences in characteristics between households with and without children.

^b^ Reflects all categories available in public use files (other categories collected but not available in public data because of small numbers).

During the survey wave just before CTC expiration (reference wave, December 29, 2021, to January 10, 2022), the prevalence of unadjusted household food insufficiency was 11.8% among all households. During the most recent survey wave available for this analysis after CTC expiration (June 29 through July 11, 2022), food insufficiency had increased to 13.6% among all households. The observed increase was larger among households with children present: in households with children, food insufficiency increased from 12.7% to 16.0% over the same period (Figure 1A and eTable 3 in the Supplement).

**Figure 2.**
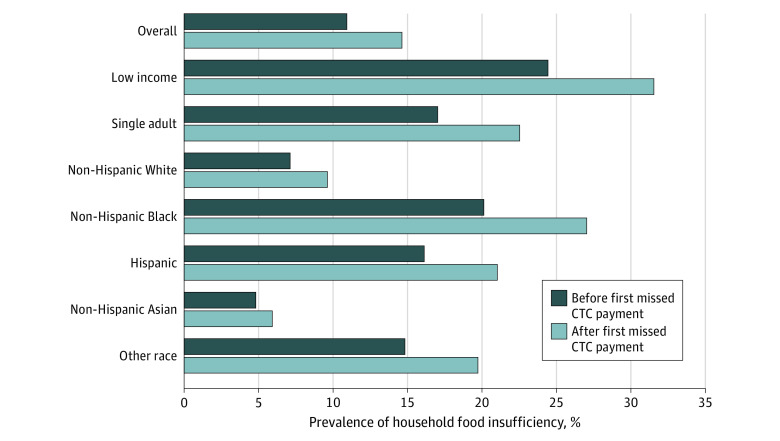
Unadjusted Food Insufficiency Before and After Missed Child Tax Credit (CTC) Payments Among Households With Children by Subgroup Unadjusted food insufficiency prevalence for the period before the first missed advance CTC payment (July 21, 2021 through January 10, 2022) is shown in dark blue. Unadjusted food insufficiency prevalence for the period after the first missed CTC payment (January 26 through July 11, 2022) is shown in light blue. Results are shown for the entire sample, for low-income households (defined as an annual household income <$35 000), single-adult households, and non-Hispanic White, Hispanic, non-Hispanic Black, non-Hispanic Asian, and another race ethnicity respondent subgroups.

Food insufficiency in households with children increased from before (July 21, 2021, to January 10, 2022) to after (January 26 to July 11, 2022) the CTC expiration in all analyzed subgroups (Figure 2). In low-income households with children, the prevalence of food insufficiency in the period before CTC expiration was 24.4%; after CTC expiration, it increased to 31.5%. Over the same period, food insufficiency increased for single-adult households and all racial and ethnic subgroups. Among racial and ethnic subgroups, food insufficiency increased the most among non-Hispanic Black, Hispanic, and another race and ethnicity households.

Our event study specification estimated that households with children present experienced increased food insufficiency in the post-CTC period, but the increases were significant only in waves 4 through 6 (April 27 to July 11, 2022) compared with households without children (Table 2 and Figure 1B). By the sixth survey wave (June 29 through July 11), food insufficiency increased by 3.2 percentage points (95% CI, 1.4-5.0 percentage points; *P* < .001), a 25% increase compared with the reference wave.

**Table 2.** Event Study Estimates of Association of Expiration of the Advance Child Tax Credit With Household Food Insufficiency

Characteristic (N = 592 044 households)	Coefficient (95% CI)	*P* value
Survey wave indicator		
July 21-August 2, 2021	–0.020 (–0.030 to –0.010)	<.001
August 4-August 16, 2021	–0.017 (–0.027 to –0.007)	.001
August 18-August 30, 2021	–0.020 (–0.030 to –0.011)	<.001
September 1-September 13, 2021	–0.014 (–0.025 to –0.004)	.007
September 15-September 27, 2021	–0.008 (–0.019 to 0.003)	.14
September 29-October 10, 2021	–0.005 (–0.015 to 0.006)	.37
December 1-December 13, 2021	–0.006 (–0.017 to 0.004)	.21
December 29, 2021-January 10, 2022	0 [Reference]	NA
January 26-February 7, 2022	–0.004 (–0.014 to 0.006)	.43
March 2-March 14, 2022	–0.002 (–0.012 to 0.007)	.65
March 30-April 11, 2022	0.011 (0.0004 to 0.022)	.04
April 27-May 9, 2022	0.010 (–0.002 to 0.021)	.10
June 1-June 13, 2022	0.006 (–0.005 to 0.017)	.30
June 29-July 11, 2022	0.007 (–0.004 to 0.018)	.19
Presence of children in household indicator	0.009 (–0.004 to 0.022)	.17
Survey wave by presence of children in household interaction		
July 21-August 2, 2021	–0.007 (–0.024 to 0.009)	.40
August 4-August 16, 2021	–0.006 (–0.022 to 0.010)	.45
August 18-August 30, 2021	0.006 (–0.010 to 0.022)	.48
September 1-September 13, 2021	–0.003 (–0.019 to 0.014)	.77
September 15-September 27, 2021	–0.003 (–0.020 to 0.014)	.73
September 29-October 10, 2021	–0.005 (–0.022 to 0.012)	.59
December 1-December 13, 2021	–0.004 (–0.020 to 0.013)	.67
December 29, 2021-January 10, 2022	0 [Reference]	NA
January 26-February 7, 2022	0.015 (–0.001 to 0.032)	.06
March 2-March 14, 2022	0.013 (–0.003 to 0.028)	.12
March 30-April 11, 2022	0.014 (–0.004 to 0.032)	.12
April 27-May 9, 2022	0.022 (0.004 to 0.040)	.02
June 1-June 13, 2022	0.022 (0.004 to 0.041)	.02
June 29-July 11, 2022	0.032 (0.014 to 0.050)	<.001
Sex at birth		
Female	0.002 (–0.001 to 0.005)	.23
Male	0 [Reference]	NA
Age group, y		
18-24	0 [Reference]	NA
25-44	0.040 (0.032 to 0.049)	<.001
45-64	0.019 (0.011 to 0.027)	<.001
Race and ethnicity		
Hispanic	0.018 (0.012 to 0.024)	<.001
Non-Hispanic		
Asian	–0.012 (–0.017 to –0.007)	<.001
Black	0.058 (0.051 to 0.065)	<.001
White	0 [Reference]	NA
Another race or ethnicity^a^	0.044 (0.035 to 0.053)	<.001
Education level		
Less than high school	0 [Reference]	NA
High school or equivalent	–0.044 (–0.057 to –0.031)	<.001
Some college or 2-y degree	–0.065 (–0.077 to –0.052)	<.001
4-y Degree or higher	–0.100 (–0.112 to –0.088)	<.001
Marital status		
Married	–0.026 (–0.031 to –0.022)	<.001
Not married	0 [Reference]	NA
Health insurance coverage		
Uninsured	0 [Reference]	NA
Public	–0.036 (–0.046 to –0.026)	<.001
Private	–0.067 (–0.074 to –0.060)	<.001
Employment for respondent in last 7 d	–0.040 (–0.044 to –0.036)	<.001
Report of unemployment insurance benefits as spending source in the last 7 d	0.016 (0.004 to 0.029)	.008
Current participation in Supplemental Nutrition Assistance Program benefits by anyone in the household	–0.001 (–0.009 to 0.007)	.82
Receipt of food aid in the last 7 d by anyone in the household	0.148 (0.137 to 0.160)	<.001
Report of Economic Impact Payment as spending source in the last 7 d	0.012 (0.006 to 0.019)	<.001
Adults in household, No.		
1	0 [Reference]	NA
2	–0.0004 (–0.006 to 0.005)	.87
≥3	0.009 (0.003 to 0.015)	.003
Children in household, No.		
0	0 [Reference]	NA
1	–0.009 (–0.016 to –0.001)	.02
2	–0.008 (–0.015 to 0.0003)	.06
≥3	0 [Omitted]	NA
Annual household income, $		
<25 000	0 [Reference]	NA
25 000-34 999	–0.068 (–0.078 to –0.058)	<.001
35 000-49 999	–0.101 (–0.110 to –0.092)	<.001
50 000-74 999	–0.141 (–0.150 to –0.133)	<.001
75 000-149 000	–0.162 (–0.171 to –0.154)	<.001
≥ 150 000	–0.156 (–0.165 to –0.148)	<.001
Missing	–0.116 (–0.126 to –0.107)	<.001

Abbreviation: NA, not applicable.

^a^ Reflects all categories available in public use files (other categories collected but not available in public data because of small numbers).

For comparison, our difference-in-differences model estimated a 2.2 percentage point increase (95% CI, 1.6-2.9 percentage points; *P* < .001) in food insufficiency among households with children over the entire period after CTC expiration compared with the entire period before CTC expiration (eTable 4 in the Supplement). In difference-in-difference analysis, the increase in food insufficiency from the pre-CTC to post-CTC expiration period in all subgroups, except non-Hispanic Asian and another race or ethnicity subgroups, was significant. The largest significant increase was among the low-income subgroup: difference-in-difference estimates found a 5.9 percentage point increase in food insufficiency among low-income households, an increase greater than any other subgroup tested, compared with a 2.2 percentage point increase for the overall sample, a 3.7 percentage point increase for single-adult households, 1.2 percentage point increase for non-Hispanic White individuals, 4.1 percentage point increase for Hispanic individuals, 3.9 percentage point increase for Non-Hispanic Black individuals, 1.3 percentage point increase for Non-Hispanic Asian individuals, and 1.7 percentage point increase for individuals of another race or ethnicity (eTable 5 in the Supplement). In all alternative models, event study coefficients remained similar and the conclusions did not change (eTable 6 in the Supplement).

## Discussion

In this cross-sectional study, we found that expiration of monthly CTC payments was associated with a 25% increase in household food insufficiency by early July 2022 compared with the period just before CTC expiration. This change suggests an erosion of positive outcomes associated with the advance CTC payments decreasing food insufficiency documented in 2021.

Although food insufficiency started increasing immediately after payment expiration in January 2022, a delay in statistically significant increases in food insufficiency in early post-CTC waves may be attributed to the onset of tax filing season. Most households with children received the second half of their advance CTC in a lump sum payment when they filed their taxes, providing an infusion of resources into household budgets. Previous research^22^ examining use of lump sum tax credits showed increased food expenditures among families with low incomes during tax season alongside other large expenditures, including bill and debt payments and purchases of durable goods. Other studies^23,24^ found that households with low incomes spend tax credits immediately upon receipt, limiting the potential effects on food purchases in months when households are not receiving payments. Furthermore, although estimates show rates of child poverty increased in early 2022 after CTC payments lapsed,^25^ those rates declined during tax season, and researchers have predicted an increase in child poverty for the remainder of the year without an additional infusion of resources for households with children.^26^

These findings are important given the robust evidence of associations between an inability to afford food and adverse child health and developmental outcomes, as well as poor health outcomes across the life span.^1^ Even brief periods of disruption in food access among households with children are associated with immediate and long-term effects on child growth, cognitive development, overall physical health, mental health, and educational attainment—all of which are associated with avoidable health and education expenditures.^27^

Consistent with previous research documenting positive outcomes of the advance CTC payment expansion, particularly for households with low incomes and children of color,^28^ our subgroup analyses found that households with low incomes experienced the greatest increases, but single-adult, non-Hispanic Black, and Hispanic households were also affected by the advance CTC expiration. Since low-income, single-adult, non-Hispanic Black, and Hispanic households with children were more likely to gain access to the CTC under the 2021 reforms and are disproportionately affected by food insufficiency, these results lend support to the observed effects being specifically attributable to the expiration of the CTC vs some other phenomenon. Furthermore, any increases in food insufficiency among these historically marginalized groups have potential implications for further exacerbating health inequities.

### Strengths and Limitations

This study is strengthened by a nationally representative sample and high-frequency, nearly real-time data on material hardships among Americans. This study is limited by being observational and its reliance on repeat cross-sectional data. Our use of event study and difference-in-differences regression models provides strong internal validity, with households not receiving the CTC (households without children present) serving as a pseudocontrol for households that did (households with children present).

## Conclusions

The expanded CTC monthly payments were previously associated with a 26% decrease in food insufficiency in 2021 among households with children. Here, we found that in the 6 months following expiration of the CTC monthly payments that their loss was associated with a 25% increase in food insufficiency. Low-income, single-adult, non-Hispanic Black, and Hispanic households experienced greater increases than the overall sample, suggesting implications of deepening inequities linked to the policy expiration. Without further Congressional action to extend the expanded CTC and reinstate monthly payments, reductions in food insufficiency, poverty, and inequity following the advance CTC payment introduction in 2021 may continue to erode.

## Notes

Supplement. **eTable 1.** Implications of ARPA Changes to the CTC **eTable 2.** Statistical Test of Parallel Trends in Household Food Insufficiency Between Households With and Without Children Present in the Period Prior to Advance Child Tax Credit Payment Expiration (July 21, 2021, through January 10, 2021) **eTable 3.** Household Food Insufficiency Overall and by Subgroup and Presence of Children in the Household, Census Bureau Household Pulse Survey, July 2021 to July 2022 **eTable 4.** Difference-in-Difference Estimates of Expiration of Advance Child Tax Credit Payments on Household Food Insufficiency **eTable 5.** Difference-in-Difference Estimates of Expiration of Advance Child Tax Credit Payments on Household Food Insufficiency: Coefficients for Post CTC Expiration × Presence of Children by Subgroup **eTable 6.** Event Study Coefficients for Survey Wave × Presence of Children in Household Interaction: Alternative Model Specifications
