# Supplementary material for: Association of the Expiration of Child Tax Credit Advance Payments With Food Insufficiency in US Households
Source: JAMA Netw Open. 2022 Oct 21;5(10):e2234438. doi: 10.1001/jamanetworkopen.2022.34438 (PMC9587477; doi:10.1001/jamanetworkopen.2022.34438)
Supplement: Supplement. — eTable 1. Implications of ARPA Changes to the CTC eTable 2. Statistical Test of Parallel Trends in Household Food Insufficiency Between Households With and Without Children Present in the Period Prior to Advance Child Tax Credit Payment Expiration (July 21, 2021, through January 10, 2021) eTable 3. Household Food Insufficiency Overall and by Subgroup and Presence of Children in the Household, Census Bureau Household Pulse Survey, July 2021 to July 2022 eTable 4. Difference-in-Difference Estimates of Expiration of Advance Child Tax Credit Payments on Household Food Insufficiency eTable 5. Difference-in-Difference Estimates of Expiration of Advance Child Tax Credit Payments on Household Food Insufficiency: Coefficients for Post CTC Expiration × Presence of Children by Subgroup eTable 6. Event Study Coefficients for Survey Wave × Presence of Children in Household Interaction: Alternative Model Specifications [file jamanetwopen-e2234438-s001.pdf]

Supplemental Online Content

Bovell-Ammon A, McCann NC, Mulugeta M, Ettinger de Cuba S, Raifman J, Shafer P. Association of the expiration of Child Tax Credit advance payments with food insufficiency in US households. *JAMA Netw Open*. 2022;5(10):e2234438. doi:10.1001/jamanetworkopen.2022.34438

eTable 1. Implications of ARPA Changes to the CTC

eTable 2. Statistical Test of Parallel Trends in Household Food Insufficiency Between Households With and Without Children Present in the Period Prior to Advance Child Tax Credit Payment Expiration (July 21, 2021, through January 10, 2021)

eTable 3. Household Food Insufficiency Overall and by Subgroup and Presence of Children in the Household, Census Bureau Household Pulse Survey, July 2021 to July 2022

eTable 4. Difference-in-Difference Estimates of Expiration of Advance Child Tax Credit Payments on Household Food Insufficiency

eTable 5. Difference-in-Difference Estimates of Expiration of Advance Child Tax Credit Payments on Household Food Insufficiency: Coefficients for Post CTC Expiration × Presence of Children by Subgroup

eTable 6. Event Study Coefficients for Survey Wave × Presence of Children in Household Interaction: Alternative Model Specifications

This supplemental material has been provided by the authors to give readers additional information about their work.

© 2022 Bovell-Ammon A et al. *JAMA Network Open.*

eTable 1. Implications of ARPA Changes to the CTC

**Pre-ARPA Structure of the CTC**	**ARPA change to the CTC**	**Implication**
Partially-refundable credit after $2,500 of earnings	Fully refundable credit beginning with $0 in earnings	Pre-ARPA expansion of the CTC, one-third of children lived in households with incomes too low to claim the full CTC. The ARPA expansion made the full value of the credit available to these households without changing phase-out eligibility for higher income households.
$2,000 per child 0-16 annually	$3,600 per child 0-<6 annually<br>$3,000 per child 6-17 annually	ARPA boosted the value of the credit for all children, created a higher value credit for young children, and increased age eligibility to age 17.
Lump sum, once per year payment at tax filing	Advance monthly payment for 6 months	ARPA enabled half of the value of the CTC to be paid in advance, monthly installments between July-December 2021. The remainder of the credit could be claimed by taxpayers filing their 2021 tax returns.

© 2022 Bovell-Ammon A et al. *JAMA Network Open.*

eTable 2. Statistical Test of Parallel Trends in Household Food Insufficiency Between Households With and Without Children Present in the Period Prior to Advance Child Tax Credit Payment Expiration (July 21, 2021, through January 10, 2021)

	**Naïve model**		**Fully-adjusted Model**	
	Coefficient (95% CI)	*P-value*	*Coefficient (95% CI)*	*P-value*
Presence of children x continuous time variable indicator	0.001 (-0.001, 0.003)	.238	0.0004 (-0.001, 0.002)	.657

Weighted using household survey weights divided by number of waves

Fully adjusted model controls for age, sex at birth, race/ethnicity, education level, number of adults in household, number of children in household, annual household income, employment in last 7 days, use of unemployment insurance for spending/funds, use of Economic Impact Payments for spending/funds, Supplemental Nutrition Assistance Program participation, receipt of food aid, health insurance coverage, and state fixed effects

Source: Authors' analysis of Household Pulse Survey data from the Census Bureau, July 2021 to July 2022

© 2022 Bovell-Ammon A et al. *JAMA Network Open.*

eTable 3. Household Food Insufficiency Overall and by Subgroup and Presence of Children in the Household, Census Bureau Household Pulse Survey, July 2021 to July 2022

**Sample, % (95% CI)**	**7/21-8/2**	**8/4-8/16**	**8/18-8/30**	**9/1-9/13**	**9/15-9/27**	**9/29-10/11**	**12/1-12/13**	**12/29/21-1/10/22**	**1/26-2/7**	**3/2-3/14**	**3/30-4/11**	**4/27-5/9**	**6/1-6/13**	**6/29-7/11**
Full sample														
Overall	9.6 (9.0, 10.2)	9.5 (8.8, 10.1)	9.7 (9.1, 10.3)	9.9 (9.2, 10.6)	10.5 (9.8, 11.2)	10.7 (10.0, 11.2)	11.0 (10.3, 11.6)	11.8 (11.2, 12.4)	11.7 (11.1, 12.3)	11.7 (11.2, 12.3)	13.0 (12.3, 13.7)	13.2 (12.5, 14.0)	12.7 (11.9, 13.4)	13.3 (12.6, 14.0)
Low-income	22.8 (21.0, 24.7)	24.5 (22.5, 26.5)	23.0 (21.2, 24.8)	22.3 (20.4, 24.3)	24.5 (22.4, 26.6)	25.0 (23.0, 26.9)	24.5 (22.7, 26.2)	25.5 (23.9, 27.0)	25.2 (23.5, 26.8)	24.7 (23.2, 26.3)	28.1 (26.1, 30.1)	29.0 (27.0, 31.1)	28.3 (26.0, 30.5)	30.9 (28.8, 32.9)
Single-adult	13.2 (11.7, 14.8)	15.6 (13.8, 17.4)	13.2 (11.9, 14.5)	12.3 (10.8, 13.9)	13.2 11.5, 15.0)	15.9 (14.1, 17.6)	14.7 (13.2, 16.2)	16.1 (14.7, 17.5)	15.2 (13.8, 16.6)	16.1 (14.7, 17.5)	17.7 (16.0, 19.3)	18.6 (16.7-20.4)	17.8 (15.8, 19.8)	17.0 (15.4, 18.6)
Non-Hispanic White	7.1 (6.5, 7.7)	7.0 (6.5, 7.6)	7.3 (6.8, 7.9)	7.3 (6.7, 8.0)	7.7 (7.0, 8.3)	7.4 (6.8, 8.1)	8.4 (7.8, 9.1)	8.2 (7.7, 8.8)	8.6 (8.0, 9.2)	8.8 (8.2, 9.4)	9.3 (8.6, 9.9)	9.5 (8.8, 10.2)	9.1 (8.5, 9.7)	9.5 (8.8, 10.2)
Hispanic	12.0 (10.2, 13.9)	13.8 (11.9, 15.8)	13.7 (11.7, 15.7)	15.2 (12.8, 17.6)	16.0 (13.2, 18.8)	16.7 (14.3, 19.1)	15.7 (13.6, 17.9)	19.1 (16.9, 21.3)	18.2 (16.1, 20.4)	16.3 (14.5, 18.2)	21.1 (18.2, 24.0)	20.8 (18.1, 23.5)	17.8 (15.0, 20.5)	16.9 (14.8, 19.0)
Non-Hispanic Black	19.1 (16.4, 21.8)	17.8 (15.1, 20.5)	17.7 (15.2, 20.2)	16.2 (14.0, 18.4)	19.9 (17.3, 24.5)	19.2 (16.6, 21.9)	18.6 (16.2, 21.1)	20.8 (18.6, 23.0)	20.4 (18.1, 22.7)	21.5 (19.4, 23.6)	23.8 (21.1, 26.4)	22.2 (19.7, 24.8)	24.8 (21.8, 27.8)	28.2 (25.4, 31.0)
Non-Hispanic Asian	4.9 (2.7, 7.0)	3.5 (2.3, 4.7)	4.4 (2.6, 6.2)	4.4 (2.8, 5.9)	4.8 (3.0, 6.5)	4.3 (2.7, 6.0)	5.2 (3.5, 6.9)	5.8 (4.0, 7.6)	3.8 (2.7, 4.9)	3.9 (2.9, 5.0)	5.0 (3.1, 6.9)	5.6 (3.8, 7.5)	6.0 (4.2, 7.8)	5.9 (3.4, 8.4)
Another race/ethnicity	15.1 (11.9, 18.3)	11.9 (9.3, 14.5)	12.4 (9.9, 14.8)	16.6 (12.3, 20.9)	12.6 (9.9, 15.2)	18.0 (14.7, 21.4)	15.4 (12.4, 18.5)	17.1 (14.3, 19.8)	17.9 (15.1, 20.7)	18.5 (15.3, 21.7)	17.8 (14.3, 21.3)	22.7 (18.7, 26.7)	17.4 (14.5, 20.3)	19.4 (15.3, 23.4)
Households with children														
Overall	9.9 (9.0, 10.9)	9.6 (8.8, 10.5)	10.8 (9.7, 11.8)	10.5 (9.4, 11.5)	10.8 (9.7, 11.9)	11.0 (9.9, 12.1)	11.9 (10.9, 13.0)	12.7 (11.8, 13.7)	13.6 (12.6, 14.6)	13.4 (12.5, 14.3)	14.5 (13.2, 15.8)	15.2 (14.0, 16.4)	14.8 (13.6, 16.0)	16.0 (14.8, 17.2)

© 2022 Bovell-Ammon A et al. *JAMA Network Open.*

Low-income	21.6 (18.4, 24.8)	23.4 (20.5, 26.3)	24.6 (21.3, 27.9)	22.3 (19.3, 25.2)	24.8 (21.6, 28.0)	24.4 (21.0, 27.8)	25.3 (22.3, 28.2)	27.9 (25.3, 30.5)	29.6 (26.7, 32.4)	27.6 (24.9, 30.3)	31.9 (28.2, 35.6)	33.1 (29.4, 36.8)	32.7 (28.9, 36.4)	34.8 (31.4, 38.2)
Single-adult	16.5 (13.0, 20.0)	18.6 (15.5, 21.7)	17.6 (14.7, 20.5)	14.0 (11.5, 16.4)	15.7 (12.7, 18.6)	15.9 (12.7, 19.1)	17.6 (14.5, 20.7)	20.0 (17.2, 22.8)	22.0 (18.8, 25.2)	21.1 (18.3, 23.8)	22.0 (18.6, 25.4)	25.2 (21.1, 29.2)	23.0 (20.0, 26.1)	22.0 (18.8, 25.1)
Non-Hispanic White	6.5 (5.7, 7.4)	6.8 (5.9, 7.8)	7.2 (6.3, 8.2)	6.2 (5.4, 7.0)	6.9 (6.0, 7.8)	7.3 (6.2, 8.4)	7.5 (6.5, 8.5)	8.4 (7.4, 9.4)	9.6 (8.6, 10.6)	9.2 (8.2, 10.2)	9.1 (8.0, 10.2)	9.6 (8.4, 10.8)	9.8 (8.7, 10.9)	10.4 (9.1, 11.7)
Hispanic	12.3 (9.6, 15.0)	13.7 (11.2, 16.3)	15.8 (12.5, 19.0)	17.6 (14.1, 21.2)	16.9 (12.9, 20.9)	16.7 (13.2, 20.1)	17.0 (14.0, 19.9)	18.6 (15.8, 21.4)	19.0 (16.2, 21.8)	18.5 (15.8, 21.1)	25.4 (20.6, 30.2)	23.5 (19.5, 27.4)	19.5 (16.3, 22.7)	20.7 (17.5, 24.0)
Non-Hispanic Black	21.1 (17.2, 25.0)	16.8 (13.9, 19.7)	19.5 (15.4, 23.6)	18.4 (15.1, 21.6)	20.8 (17.4, 24.1)	19.5 (15.6, 23.4)	23.8 (19.9, 27.7)	21.1 (18.1, 24.1)	24.0 (20.3, 27.7)	23.3 (20.3, 26.3)	25.4 (21.6, 29.2)	27.2 (23.3, 31.1)	28.5 (23.8, 33.3)	33.8 (29.7, 37.9)
Non-Hispanic Asian	3.5 (1.9, 5.0)	3.9 (1.9, 6.0)	4.7 (1.3, 8.2)	3.8 (1.9, 5.6)	4.8 (2.4, 7.3)	5.0 (1.9, 8.1)	4.7 (2.5, 6.8)	7.4 (4.1, 10.8)	4.0 (2.3, 5.6)	4.9 (3.1, 6.7)	6.2 (2.6, 9.9)	6.3 (3.8, 8.8)	7.8 (4.7, 10.9)	6.4 (2.7, 10.0)
Another race/ethnicity	16.6 (11.3, 21.9)	12.1 (8.3, 15.9)	13.2 (9.7, 16.7)	15.6 (7.6, 23.5)	11.9 (7.7, 16.1)	15.9 (11.7, 20.1)	15.7 (10.8, 20.7)	17.0 (13.6, 20.5)	18.9 (14.5, 23.3)	19.6 (15.1, 24.2)	15.6 (11.3, 20.0)	24.1 (18.5, 29.7)	21.6 (16.6, 26.5)	18.2 (13.3, 23.1)
Households without children														
Overall	9.3 (8.5, 10.1)	9.3 (8.50, 10.2)	8.9 (8.2, 9.5)	9.5 (8.6, 10.3)	10.3 (9.4, 11.3)	10.4 (9.5, 11.2)	10.3 (9.5, 11.0)	11.1 (10.3, 11.8)	10.4 (9.6, 11.1)	10.5 (9.8, 11.2)	12.0 (11.1, 12.8)	11.9 (11.0, 12.8)	11.2 (10.2, 12.1)	11.5 (10.6, 12.3)
Low-income	23.6 (21.3, 26.0)	25.2 (22.6, 27.9)	22.0 (20.0, 24.0)	22.4 (19.8, 24.9)	24.4 (21.6, 27.1)	25.3 (23.0, 27.7)	23.9 (21.7, 26.1)	23.9 (21.9, 25.9)	22.4 (20.4, 24.4)	22.9 (21.0, 24.8)	26.0 (23.7, 28.2)	26.9 (24.4, 29.4)	25.7 (22.8, 28.5)	28.6 (26.1, 31.2)
Single-adult	11.9 (10.2, 13.6)	14.2 (11.9, 16.4)	11.5 (10.1, 12.8)	11.7 (9.7, 13.6)	12.2 (10.0, 14.3)	15.9 (13.8, 18.0)	13.6 (12.0, 15.3)	14.5 (12.9, 16.1)	12.5 (11.1, 14.0)	14.1 (12.5, 15.7)	16.0 (14.2, 17.8)	15.9 (14.0, 17.9)	15.8 (13.2, 18.3)	15.1 (13.3, 16.9)
Non-Hispanic White	7.4 (6.6, 8.2)	7.2 (6.4, 7.9)	7.4 (6.7, 8.1)	8.0 (7.1, 9.0)	8.1 (7.2, 9.0)	7.6 (6.8, 8.3)	9.0 (8.2, 9.8)	8.1 (7.4, 8.8)	8.0 (7.3, 8.7)	8.6 (7.8, 9.3)	9.3 (8.5, 10.2)	9.4 (8.5, 10.4)	8.7 (7.9, 9.5)	9.0 (8.1, 9.78

© 2022 Bovell-Ammon A et al. *JAMA Network Open.*

Hispanic	11.8 (9.4, 14.2)	14.0 (11.0, 16.9)	11.5 (9.3, 13.7)	12.7 (9.5, 16.0)	15.0 (11.1, 18.9)	16.7 (13.4, 20.0)	14.2 (11.1, 17.3)	19.7 (16.2, 23.1)	17.4 (14.1, 20.8)	14.0 (11.5, 16.5)	17.0 (14.0, 20.1)	18.3 (14.7, 21.9)	16.0 (11.5, 20.6)	13.4 (10.7, 16.1)
Non-Hispanic Black	17.3 (13.6, 21.0)	18.7 (14.3, 23.1)	16.1 (13.2, 18.9)	14.0 (11.1, 16.8)	19.2 (15.3, 23.0)	19.0 (15.4, 22.5)	14.1 (11.2, 17.0)	20.5 (17.3, 23.7)	17.0 (14.2, 19.8)	19.7 (16.7, 22.7)	22.5 (18.8, 26.1)	18.1 14.6, 21.5)	21.3 (17.7, 25.0)	23.2 (19.6, 26.8)
Non-Hispanic Asian	6.0 (2.3, 9.8)	3.1 (1.7, 4.5)	4.1 (2.4, 5.9)	4.9 (2.5, 7.3)	4.7 (2.2, 7.2)	3.8 (2.3, 5.3)	5.6 (3.2, 8.1)	4.3 (2.7, 5.8)	3.6 (2.1, 5.2)	3.2 (1.9, 4.5)	4.0 (2.4, 5.5)	5.1 (2.5, 7.7)	4.6 (2.5, 6.6)	5.6 (2.1, 9.0)
Another race/ethnicity	14.0 (10.0, 18.0)	11.7 (8.2, 15.2)	11.6 (8.2, 15.1)	17.5 (13.3, 21.8)	13.1 (9.7, 16.4)	20.0 (14.9, 25.1)	15.1 (11.4, 18.9)	17.1 (13.0, 21.2)	17.1 (13.5, 20.8)	17.7 (13.2, 22.2)	19.4 (14.3, 24.5)	21.7 (16.1, 27.3)	14.4 (11.0, 17.9)	20.1 (14.3, 26.0)

Weighted using household survey weights divided by number of waves

Source: Authors' analysis of Household Pulse Survey data from the Census Bureau, July 2021 to July 2022

© 2022 Bovell-Ammon A et al. *JAMA Network Open.*

eTable 4. Difference-in-Difference Estimates of Expiration of Advance Child Tax Credit Payments on Household Food Insufficiency

**Characteristic**		
	Coefficient (95% confidence interval)	*P-value*
Post-period indicator	0.034 (0.025, -0.043)	<.001
Presence of children in household indicator	0.007 (-0.001, -0.014)	.090
Post period x presence of children in household indicator	0.022 (0.016, -0.029)	<.001
Sex at birth		
Female	0.002 (-0.001, -0.005)	.234
Male	0 [reference]	NA
Age group		
18-24 years of age	0 [reference]	NA
25-44 years of age	0.040 (0.032, -0.049)	<.001
45-64 years of age	0.019 (0.011, -0.027)	<.001
Race/ethnicity		
Non-Hispanic White	0 [reference]	NA
Hispanic	0.018 (0.012, -0.024)	<.001
Non-Hispanic Black	0.058 (0.051, -0.065)	<.001
Non-Hispanic Asian	-0.012 (-0.017, -0.007)	<.001
Another race/ethnicity	0.044 (0.035, -0.053)	<.001
Education		
Less than high school	0 [reference]	NA
High school or equivalent	-0.044 (-0.057, -0.031)	<.001
Some college or 2-year degree	-0.065 (-0.077, -0.052)	<.001
4-year degree or more	-0.100 (-0.112, -0.088)	<.001
Marital status		
Married	-0.026 (-0.031, -0.022)	<.001
Not married	0 [reference]	NA
Health insurance coverage		
Uninsured	0 [reference]	NA
Public	-0.036 (-0.046, -0.026)	<.001
Private	-0.067 (-0.074, -0.060)	<.001
Respondent employed in the last 7 days	-0.040 (-0.044, -0.036)	<.001

© 2022 Bovell-Ammon A et al. *JAMA Network Open.*

Report of unemployment insurance benefits as spending source in the last 7 days	0.016 (0.004, 0.029)	.008
Current participation in Supplemental Nutrition Assistance Program benefits by anyone in the household	-0.001 (-0.009, 0.007)	.817
Receipt of food aid in the last 7 days by anyone in the household	0.148 (0.137, 0.160)	<.001
Report of Economic Impact Payment as spending source in the last 7 days	0.012 (0.006, 0.019)	<.001
Number of adults in household		
1	0 [reference]	NA
2	-0.0004 (-0.006, 0.005)	.876
3 or more	0.009 (0.003, 0.015)	.003
Number of children in household		
0	0 [reference]	NA
1	-0.009 (-0.016, -0.001)	.021
2	-0.008 (-0.016, 0.0003)	.058
3 or more	0 [omitted]	NA
Household income		
<$25,000	0 [reference]	NA
$25,000-34,999	-0.068 (-0.078, -0.058)	<.001
$35,000-49,999	-0.101 (-0.110, -0.092)	<.001
$50,000-74,999	-0.141 (-0.150, -0.133)	<.001
$75,000-149,999	-0.162 (-0.171, -0.154)	<.001
>=$150,000	-0.156 (-0.165, -0.148)	<.001
Missing	-0.116 (-0.126, -0.107)	<.001

Model also includes state and survey wave fixed effects and a constant term, weighted using household survey weights divided by the number of waves

Source: Authors' analysis of Household Pulse Survey data from the Census Bureau, July 2021 to July 2022

© 2022 Bovell-Ammon A et al. *JAMA Network Open.*

eTable 5. Difference-in-Difference Estimates of Expiration of Advance Child Tax Credit Payments on Household Food Insufficiency: Coefficients for Post CTC Expiration × Presence of Children by Subgroup

**Characteristic: post CTC expiration indicator x presence of children in household interaction**	**Household food insufficiency**	
	Coefficient (95% CI)	*P-value*
Main model: whole sample (N=592,044)	0.022 (0.016, 0.029)	<.001
Low-income households (N=100,427)	0.059 (0.038, 0.080)	<.001
Single-adult households (N=125,620)	0.037 (0.018, 0.056)	<.001
Race/ethnicity: non-Hispanic White (N=425,497)	0.012 (0.006, 0.019)	<.001
Race/ethnicity: Hispanic (N=61,265)	0.041 (0.017, 0.064)	0.001
Race/ethnicity: non-Hispanic Black (N=46,704)	0.039 (0.013, 0.065)	0.004
Race/ethnicity: non-Hispanic Asian (N=34,118)	0.013 (-0.005, 0.031)	.146
Race/ethnicity: other (N=24,460)	0.017 (-0.016, 0.050)	.304

Table reports coefficient of interest: the interaction between having children in the household and being in the period after CTC expiration (January 26th through July 11th, 2022). Full model results are not presented. Each model controls for the covariates age, sex at birth, race/ethnicity, education level, number of adults in household, number of children in household, annual household income, employment in last 7 days, use of unemployment insurance for spending/funds, use of Economic Impact Payments for spending/funds, Supplemental Nutrition Assistance Program participation, receipt of food aid, health insurance coverage, and state fixed effects

© 2022 Bovell-Ammon A et al. *JAMA Network Open.*

eTable 6. Event Study Coefficients for Survey Wave × Presence of Children in Household Interaction: Alternative Model Specifications

**Characteristic: survey wave x presence of children in household interaction**	**Household food insufficiency**	
	Coefficient (95% CI)	*P-value*
Main model		
July 21 – August 2, 2021	-0.007 (-0.024, 0.009)	.397
August 4 – August 16, 2021	-0.006 (-0.022, 0.010)	.448
August 18 – August 30, 2021	0.006 (-0.010, 0.022)	.475
September 1 – September 13, 2021	-0.003 (-0.019, 0.014)	.767
September 15 – September 27, 2021	-0.003 (-0.020, 0.014)	.729
September 29 – October 10, 2021	-0.005 (-0.022, 0.012)	.587
December 1 – December 13, 2021	-0.004 (-0.020, 0.013)	.671
December 29, 2021 – January 10, 2022	0 [reference]	NA
January 26 – February 7, 2022	0.015 (-0.001, 0.032)	.064
March 2 – March 14, 2022	0.013 (-0.003, 0.028)	.115
March 30 – April 11, 2022	0.014 (-0.004, 0.032)	.122
April 27 – May 9, 2022	0.022 (0.004, 0.040)	.017
June 1 – June 13, 2022	0.022 (0.004, 0.041)	.016
June 29 – July 11, 2022	0.032 (0.014, 0.050)	<.001
Alternative model 1: clustered standard errors by state		
July 21 – August 2, 2021	-0.007 (-0.022, 0.008)	.356
August 4 – August 16, 2021	-0.006 (-0.019, 0.007)	.353
August 18 – August 30, 2021	0.006 (-0.010, 0.021)	.443
September 1 – September 13, 2021	-0.003 (-0.014, 0.009)	.660
September 15 – September 27, 2021	-0.003 (-0.016, 0.009)	.623
September 29 – October 10, 2021	-0.005 (-0.022, 0.013)	.582
December 1 – December 13, 2021	-0.004 (-0.017, 0.009)	.581
December 29, 2021 – January 10, 2022	0 [reference]	NA
January 26 – February 7, 2022	0.015 (0.008, 0.023)	<.001
March 2 – March 14, 2022	0.013 (-0.0004, 0.026)	.057
March 30 – April 11, 2022	0.014 (-0.008, 0.036)	.201
April 27 – May 9, 2022	0.022 (0.001, 0.043)	.040
June 1 – June 13, 2022	0.022 (0.005, 0.040)	.013
June 29 – July 11, 2022	0.032 (0.019, 0.046)	<.001

© 2022 Bovell-Ammon A et al. *JAMA Network Open.*

Alternative model 2: pre-period shortened to period before federal eviction moratorium and enhanced unemployment benefit expiration		
September 29 – October 10, 2021	-0.005 (-0.022, 0.013)	.607
December 1 – December 13, 2021	-0.004 (-0.020, 0.013)	.673
December 29, 2021 – January 10, 2022	0 [reference]	NA
January 26 – February 7, 2022	0.015(-0.001, 0.032)	.065
March 2 – March 14, 2022	0.013 (-0.003, 0.029)	.111
March 30 – April 11, 2022	0.015 (-0.003, 0.033)	.110
April 27 – May 9, 2022	0.022 (0.004, 0.040)	.015
June 1 – June 13, 2022	0.023 (0.004, 0.041)	.016
June 29 – July 11, 2022	0.033 (0.015, 0.050)	<.001
Alternative model 3: controlling for interaction between presence of children in household and unemployment insurance		
July 21 – August 2, 2021	-0.006 (-0.022, 0.011)	.485
August 4 – August 16, 2021	-0.005 (-0.021, 0.011)	.525
August 18 – August 30, 2021	0.007 (-0.009, 0.023)	.410
September 1 – September 13, 2021	-0.002 (-0.018, 0.015)	.854
September 15 – September 27, 2021	-0.002 (-0.020, 0.015)	.792
September 29 – October 10, 2021	-0.004 (-0.022, 0.013)	.621
December 1 – December 13, 2021	-0.004 (-0.020, 0.013)	.675
December 29, 2021 – January 10, 2022	0 [reference]	NA
January 26 – February 7, 2022	0.015 (-0.001, 0.032)	.065
March 2 – March 14, 2022	0.013 (-0.003, 0.028)	.116
March 30 – April 11, 2022	0.014 (-0.004, 0.032)	.124
April 27 – May 9, 2022	0.022 (0.004, 0.040)	.018
June 1 – June 13, 2022	0.022 (0.004, 0.041)	.017
June 29 – July 11, 2022	0.032 (0.014, 0.050)	<.001
Alternative model 4: fully saturated, controlling for interaction between presence of children in household and all other included covariates		
July 21 – August 2, 2021	-0.006 (-0.023, 0.010)	.461
August 4 – August 16, 2021	-0.005 (-0.021, 0.011)	.503
August 18 – August 30, 2021	0.006 (-0.010, 0.022)	.479
September 1 – September 13, 2021	-0.002 (-0.019, 0.014)	.777
September 15 – September 27, 2021	-0.003 (-0.021, 0.014)	.696
September 29 – October 10, 2021	-0.005 (-0.022, 0.012)	.571
December 1 – December 13, 2021	-0.003 (-0.020, 0.014)	.720
December 29, 2021 – January 10, 2022	0 [reference]	NA
January 26 – February 7, 2022	0.015 (-0.001, 0.032)	.065

© 2022 Bovell-Ammon A et al. *JAMA Network Open.*

March 2 – March 14, 2022	0.013 (-0.003, 0.028)	.117
March 30 – April 11, 2022	0.014 (-0.004, 0.032)	.117
April 27 – May 9, 2022	0.021 (0.003, 0.039)	.020
June 1 – June 13, 2022	0.022 (0.004, 0.040)	.017
June 29 – July 11, 2022	0.032 (0.014, 0.049)	<.001

Table reports coefficient of interest: the interaction between having children in the household and survey wave. Full model results are not presented. Each model controls for the same covariates included in the main model: age, sex at birth, race/ethnicity, education level, number of adults in household, number of children in household, annual household income, employment in last 7 days, use of unemployment insurance for spending/funds, use of Economic Impact Payments for spending/funds, Supplemental Nutrition Assistance Program participation, receipt of food aid, health insurance coverage, and state fixed effects

© 2022 Bovell-Ammon A et al. *JAMA Network Open.*
